# Supplementary material for: NF-κB restrains nutrient-dependent transcription programs through chromatin modulation in Drosophila
Source: Nucleic Acids Res. 2026 Jan 15;54(2):gkaf1530. doi: 10.1093/nar/gkaf1530 (PMC12805896; doi:10.1093/nar/gkaf1530)
Supplement: gkaf1530_Supplemental_File [file gkaf1530_supplemental_file.pdf]

Supplemental information

NF-κB restrains nutrient-dependent transcription programs through chromatin modulation in *Drosophila*

Xiangshuo Kong[1,2], Conghui Li[1] and Jason Karpac[1,2]*

[1]Department of Biology, Texas A&M University, College Station, TX, 77843, USA

[2]Department of Cell Biology and Genetics, College of Medicine, Texas A&M University, Bryan, TX, 77807, USA

*Correspondence: karpac@tamu.edu

Table S1: qRT-PCR Primers

Primer	Sequence (5’-3’)
actin5C-F	CTCGCCACTTGCGTTTACAGT
actin5C-R	TCCATATCGTCCCAGTTGGTC
Lamp1-F	ACCTATGAGGCTAGGGAGGG
Lamp1-R	GGAAAGCACAGTGGTGTCAT
Atg1-F	CACGGCACACATTCTGCTTC
Atg1-R	CAACGGCATCGCGATATTTGT
Rab40-F	GCGAGTCCTTTTGCGAACTG
Rab40-R	CCTGAAGCGACAGCACCTTA
Bmm-F	CAATAAGGGTCTGGCCAACTGGAT
Bmm-R	TAAGTCCTCCACCATTACTCTGGC
CaMKII-F	TTCACCATCGTTTTCCTGCT
CaMKII -R	GGCAGCTTGAGTCAGGTGAT
mdy-F	CGGAACTCCCTGATTCCGTT
mdy-R	AGATGATTGGGTAGCGCAAGT
ImpL2-F	AACCGCGAATCATCTACACC
ImpL2-R	GGACGATCTCCTTGTTCTCG
wdb-F	GACGTGTTCCCAGAAGGAGG
wdb-R	GCTCGCTCAGCAACCTGAAA
trbl-F	CGAGGGCCATATTCCACCAG
trbl-R	TACTGCAGTTTCGTTCTGGC
Itpr-F	AGCCAAGGAAGTAGCACCAC
Itpr-R	ACAAACAAGCCCTCCTTGCT
yellow-h-F	ATCCGGATCCCTTTGCTTCG
yellow-h-R	AGGCACCATGAACTCCTTGAA
Hgo-F	TTACCGTGCTCACTTGTCCC
Hgo-R	GCTCATGCAGTTCCTGTGGT
trh-F	CACTGCGAGCCAAGGGTATC
trh-R	CGATCAGGTCTGAGTGGCTC
Hpd-F	ATTCGTGAGATTCGCTACCATC

Hpd-R	GGCAATCCGTTTAGAAGGACATC
Tat-F	TGATTGATGTGCTCCACTCGAA
Tat-R	TGGACAAAGTGGGTGTCGTC
Faa-F	TTCCCTATCTGCGCCAGAAC
Faa-R	GTTTTGATCGGCGGGTTTCAA
Hydr2-F	TCAAGGAGTATGCCACGACG
Hydr2-R	GCATCATGACCAAGGACCCA
Oapt74D-F	GCTTGCATTCCAGATGCTCC
Oapt74D-R	GCCGACTGTATCACACCCAT
Pepck1-F	ACAAAGCAAAATTATCTCCGGCA
Pepck1-R	AGATGGGAATGCCACGGATG
Pepck2-F	TGTCATTCCTTTTAGCATGGGTC
Pepck2-R	GACGACGTATGGTGAGTCTGT
Sik3-F	CGCGATCAAATGATCCCCCT
Sik3-R	GTAGGGAACTGGCTGAGTGG
Glys-F	CGCACCCTGGGCATTTACTA
Glys-R	GAGTCCTCGTCATCCGAACC
Glyp-F	CCACTACTACTTGCTGGCCG
Glyp-R	CCACTTGGCTTGGTTCTGGT
Dg-F	GCAAAATGGAGCTAGGCGAC
Dg-R	TTGTAGGATGGTGGCAGCAG
ActinBeta-F	TGTGGTTGCCCTTAGCCTTG
ActinBeta-R	ATTGGCGCTCAGTTAGGACA
Hsp83-F	CCCTTACTGACCCCAGCAAG
Hsp83-R	GATGGTCAGAGTACCAGCCG
Usp47-F	CATCGCAGCGGAGTTCATTG
Usp47-R	CGGACACCACCGTCTTACAA
Cdc37-F	TTCGACAGCGAGATCGAAGG
Cdc37-R	AGGCCTTTAGTTCATCGGGC
step-F	GGATAAGGAACCACGCGGAA
step-R	TTGCCCTCTACCACTTTGCC

melt-F	TACTCAAGTGCGAGAACCGC
melt-R	GGATCTCGCGGTCCTTAACC
CycG-F	TCGTTCCACTTGGCCATCAA
CycG-R	AGCGGTACAACCACACTGAG
Lin28-F	CCGCACAAGAATGTGACCCA
Lin28-R	TCTTCTCTTGGGCTGCACTG
Gbs-76A-F	ATGTCCTCATGTCGCGGAAG
Gbs-76A-R	CCTCCTCGGTTGTCTGTGAT
Hr96-F	TTGGATCGCGAGCTAAACGA
Hr96-R	CGAGTGTCGTCGGGCTTAAT
Gbs-70E-F	ATCCTCCGGATCAGTGGACA
Gbs-70E-R	AGTCGCGTTCTATCTTTTGTCTAA
Pask-F	TGCGTTAATGAAGCCAAGCAC
Pask-R	GACCCTCAGCTCGTGGTTAC

Table S2: ChIP-qPCR Primers

Primer	Sequence (5’-3’)
ChIP-Atg1-F	GAAAGTGGTATTTTTCGCGC
ChIP-Atg1-R	ACGATCAGCGTAATTTCCTT
ChIP-BmmR1-F	GCTTGTTTGCGTTTGTAGGTC
ChIP-BmmR1-R	TTCGAAATTGGACAAACACG
ChIP-BmmR2-F	TGTCGCTGACAATCAAAAGC
ChIP-BmmR2-R	TTCTGGGTGGAGTTTGGAAC
ChIP-Faa-F	AAGTTCTGTGGTGGTTTCCG
ChIP-Faa-R	TATGGGTTCACCCAGCTGAT
ChIP-Tat-F	TAAAGGGCTCCAAGCTATCC
ChIP-Tat-R	TTTATGGTGCTCACCAATGG
ChIP-Hpd-F	GGAGATTTCCTGCCTGGATT
ChIP-Hpd-R	GCGGAGTATTCCGTGTGAAT

Supplementary Figures

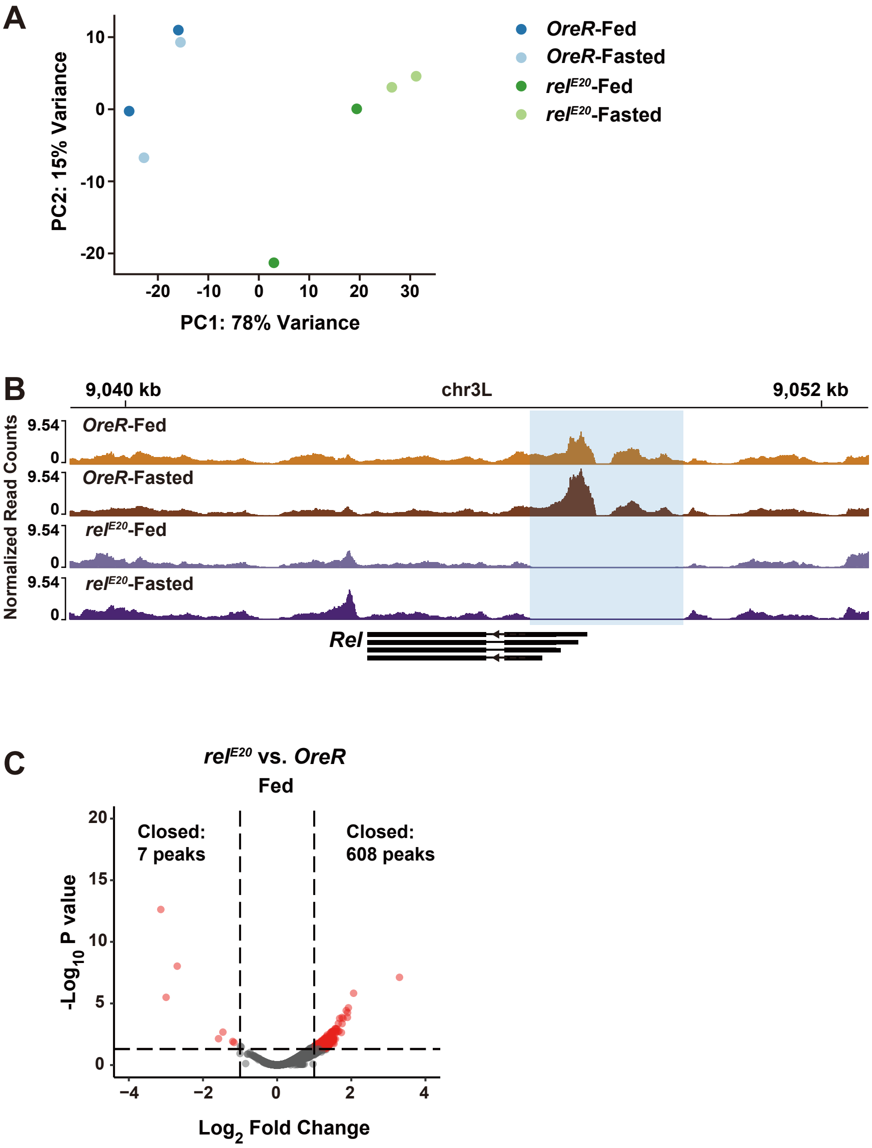

Figure S1. Landscape of DNA accessibility in *OreR* control and Relish mutant flies. Related to Figure 1.

(A) PCA of peak accessibility in all samples. Each symbol represents an independent biological replicate. PC1 (78%) and PC2 (15%). (B) Normalized chromatin accessibility profiles at *Rel* gene locus. Shaded region indicates the genomic region deleted in Relish mutant flies. (C) Volcano plot showing significantly changed peaks in rel^{E20} mutants and OreR control flies under fed conditions.

Fasted represents 20h of nutrient deprivation. All flies were 7 days old post-

eclosion.

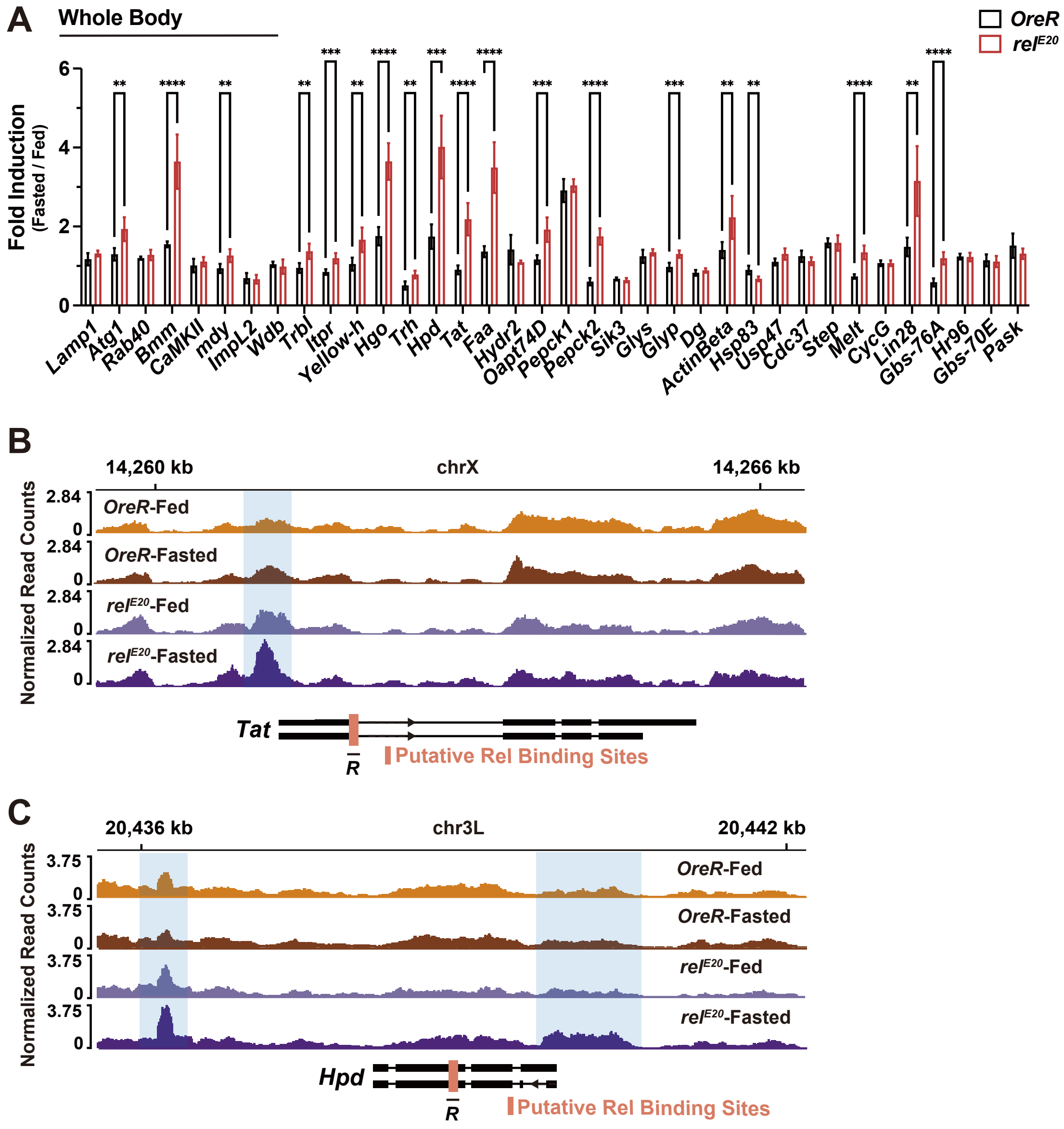

Figure S2. Relish-dependent changes in metabolic gene expression and chromatin accessibility. Related to Figure 2.

(A) Transcriptional changes (measured by qRT-PCR in the whole body of *OreR* control and Relish mutant flies, plotted as fold induction [ratio of 20 h fasted to fed] of relative expression) of distinct upregulated metabolic genes identified in Figure 1F. n = 5 replicates. (B and C) Normalized chromatin accessibility profiles at individual targeted gene loci. Shaded regions indicate chromatin peaks exhibiting uniquely increased accessibility in fasted Relish mutant flies. Red bars represent Relish binding sites predicted by JASPAR (relative profile score threshold > 75%). $\bar{R}$ represent regional target sites (and corresponding primer sets) tested in ChIP-qPCR analysis.

Bars in (A) represent mean ± SE. **p value < 0.01; ***p value < 0.001; ****p value < 0.0001. All flies were 7 days old post-eclosion.

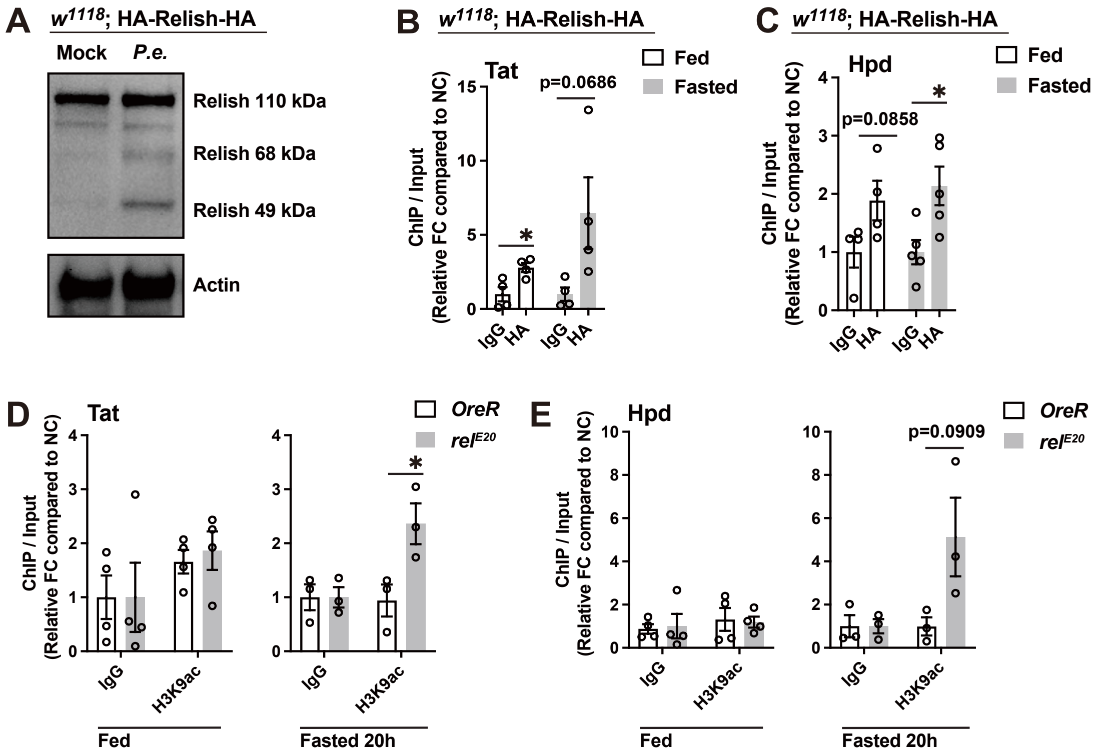

Figure S3. Relish-dependent changes in chromatin modifications. Related to Figure 2.

(A) The Relish protein and its cleaved forms upon bacterial infection. Genotype: *w1118*; HA-Relish-HA. (B and C) ChIP-qPCR analysis of Relish binding to the predicted sites in fed and fasted (20h) conditions (genotype: *w1118*; HA-Relish-HA). ChIP-qPCR analysis with normal IgG is included as a control. Plotted as fold change (FC) of indicated PCR primer sets compared to a negative control (NC) primer set. n= 4-5 biological replicates. (D and E) ChIP-PCR analysis of H3K9ac enrichment in Relish-binding regions in *OreR* control and Relish mutant genotypes before and after fasting (20 h). n = 3-4 biological replicates.

Bars in (B-E) represent mean ± SEM. *p value < 0.05. All flies were 7 days old post-eclosion.

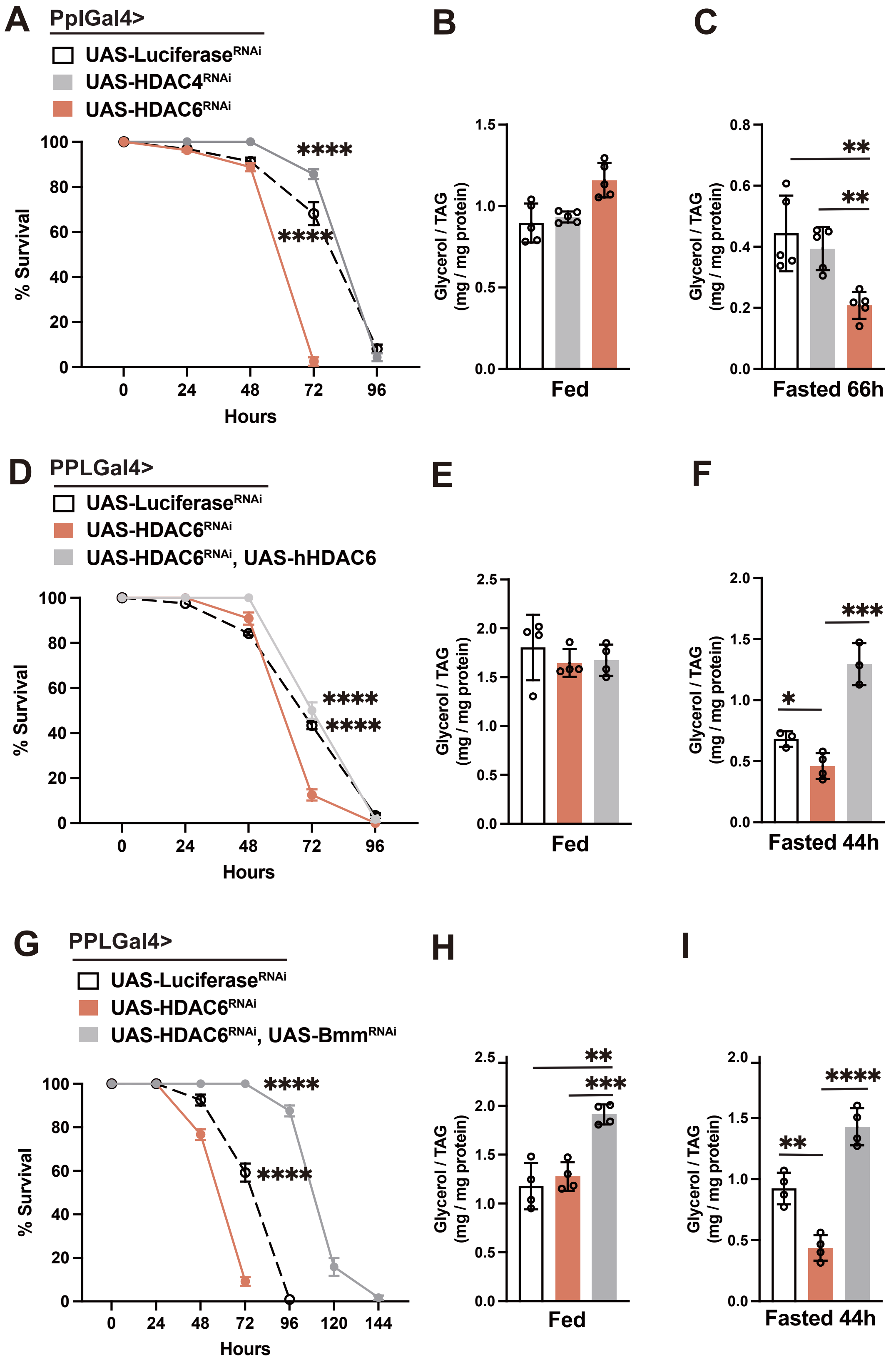

Figure S4. HDAC6-dependent changes in lipid metabolism in response to metabolic adaptation. Related to Figure 4 and 5.

(A) Starvation resistance of female flies (PplGal4/+; UAS-Luciferase^{RNAi},

PplGal4/+; UAS-HDAC4^{RNAi} or PplGal4/+; UAS-HDAC6^{RNAi}). n=8 cohorts (total 160 flies). (B and C) Total TAG levels of whole bodies during feeding and fasting conditions (n = 5 samples). (D-F) Re-expressing hHDAC6 in the fat body of HDAC6-deficient flies restores metabolic adaptation responses. (D) Starvation resistance of female flies (PplGal4/+; UAS-Luciferase^{RNAi}, PplGal4/+; UAS-HDAC6^{RNAi} or PplGal4/UAS-hHDAC6; UAS-HDAC6^{RNAi}/+). n=6 cohorts (total 120 flies). (E and F) Total TAG levels of whole bodies during feeding and fasting conditions (n = 3-4 samples). (G-I) Attenuating Bmm in the fat body of HDAC6-deficient flies restores metabolic adaptation responses. (G) Starvation resistance of female flies (PplGal4/+; UAS-Luciferase^{RNAi}, PplGal4/+; UAS-HDAC6^{RNAi} or PplGal4/+; UAS-HDAC6^{RNAi}/UAS-Bmm^{RNAi}). n=6 cohorts (total 120 flies). (H and I) Total TAG levels of whole bodies during feeding and fasting conditions (n = 3-4 samples).

Bars in (A, D and G) represent mean ± SEM, bars in (B, C, E, F, H and I) represent mean ± SE. *p value < 0.05; **p value < 0.01; ***p value < 0.001; ****p value < 0.0001. All flies were 7 days old post-eclosion.

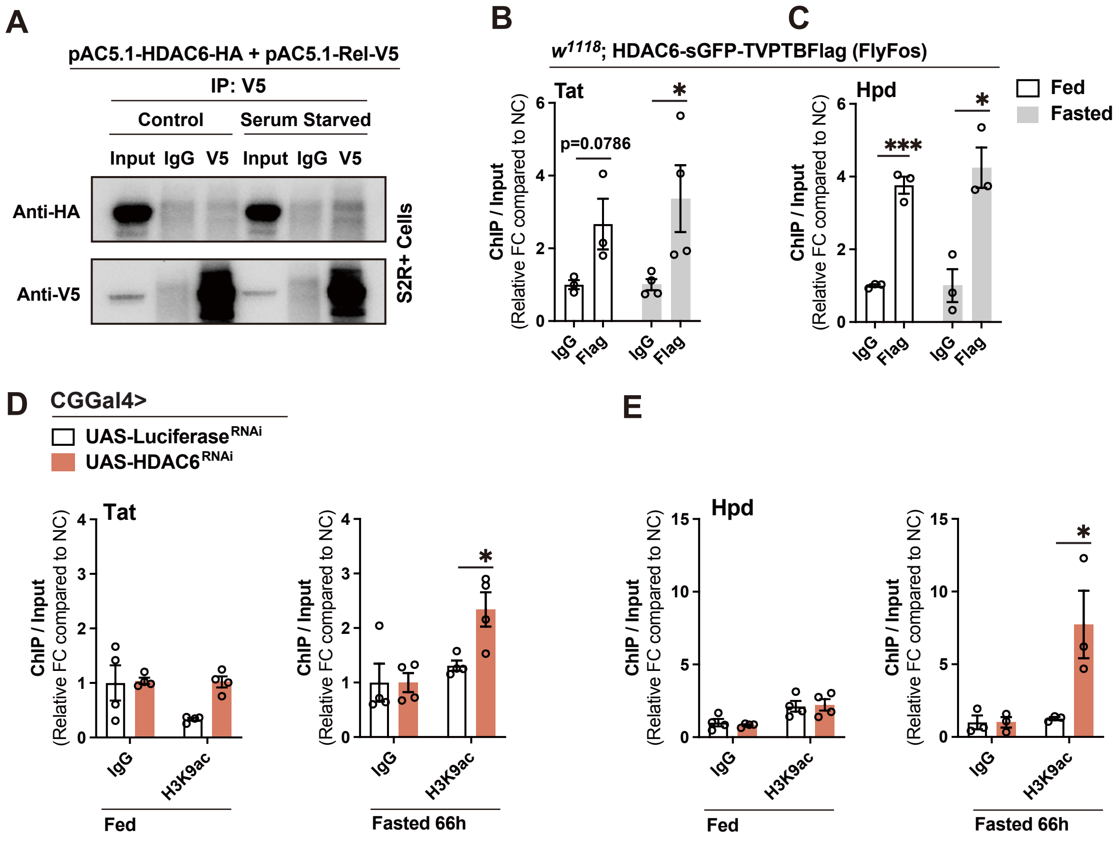

Figure S5. HDAC6-dependent changes in chromatin organization in response to metabolic adaptation. Related to Figure 6.

(A) *In vitro* interaction of Relish with HDAC6. Immunoprecipitates were prepared from lysates of S2R+ cells transfected with plasmids pAC5.1-HDAC6-HA and pAC5.1-Rel-V5. (B and C) ChIP-qPCR analysis of HDAC6 binding to the Relish-binding sites in fed and fasted (20h) conditions (genotype: *w1118*; HDAC6-sGFP-TVPTBFlag). n= 3-4 biological replicates. (D and E) ChIP-qPCR analysis of H3K9ac enrichment in Relish-binding regions in CGGal4/+; UAS-Luciferase^{RNAi} and CGGal4/+; UAS-HDAC6^{RNAi} genotypes before and after fasting (66 h). n = 3-5 biological replicates.

Bars in (B-E) represent mean ± SEM. *p value < 0.05; ***p value < 0.001. All flies were 7 days old post-eclosion.
